# Modeling the larvae dispersion of sun coral in the Brazil current off Cape Frio: A cyclonic eddy scenario

**DOI:** 10.1371/journal.pone.0295534

**Published:** 2023-12-14

**Authors:** Leandro Calado, Bernardo Cosenza, Francisco Moraes, Damián Mizrahi, Fabio C. Xavier, Daniela Batista, Sávio Calazans, Fernanda Araújo, Ricardo Coutinho

**Affiliations:** 1 Marine Biotechnology Department, Instituto de Estudos do Mar Almirante Paulo Moreira (IEAPM), Arraial do Cabo, Rio de Janeiro, Brazil; 2 Department of Oceanography, Universidade do Estado do Rio de Janeiro, Rio de Janeiro, Rio de Janeiro, Brazil; Universidade de Aveiro, PORTUGAL

## Abstract

The study aims to understand the dispersal patterns of non-indigenous *Tubastraea* spp. (Sun Coral) larvae in the Brazil Current (BC), specifically in the Cape Frio recurrent cyclonic eddy (CFE) scenario. For this, the Regional Ocean Model System was used to simulate the hydrodynamic fields in a high-resolution nested grid, where a model of lagrangian floats, in a good approximation of the larvae properties and considering massive planulation events, was coupled with surface larval release from the Campos Basin area. The simulation was representative of mesoscale features compared to similar studies, ARGO vertical profiles and a py-eddy-track algorithm was used to obtain eddy variables, such as radius, rotational and translational velocities. These parameters are fundamental to access when an eddy tends to trap or not the water, heat and plankton in its interior. CFE turned out to be highly nonlinear, with a strong tendency to trap larvae in its core, acting as a dispersal constrictor when compared with the organisms in the axis of the higher speed of BC. A strong negative correlation (-0.75) was found between the days that larvae were inside the eddy and their distance from the origin. None of the 48,000 larvae released during simulated experiment a 16-day spawning event reached the coast. There are two different patterns for the dispersal, one along the shelf break and another, with higher larval density, off from the 1000 m isobath. The CFE’s presence allows larvae to remain in the same region for longer periods, although in offshore areas. Therefore, as there is considerable availability of fixed substrates on oil rig structures, larvae could settle on them resulting in a possible inter-platforms connectivity between populations of *Tubastraea* spp. Also, regions in the CFE that present downward vertical velocities (downwelling), may move young larvae to depths of about 60 m suggesting that subsurface colonizations are possible due to specific dynamics of propagating cyclonic eddies. So, identifying the main factors that affect the dispersion of propagules is essential to subsidize management policies for controlling bioinvasion associated with exploitation of hydrocarbon resources in offshore areas.

## Introduction

**Fig 1:**
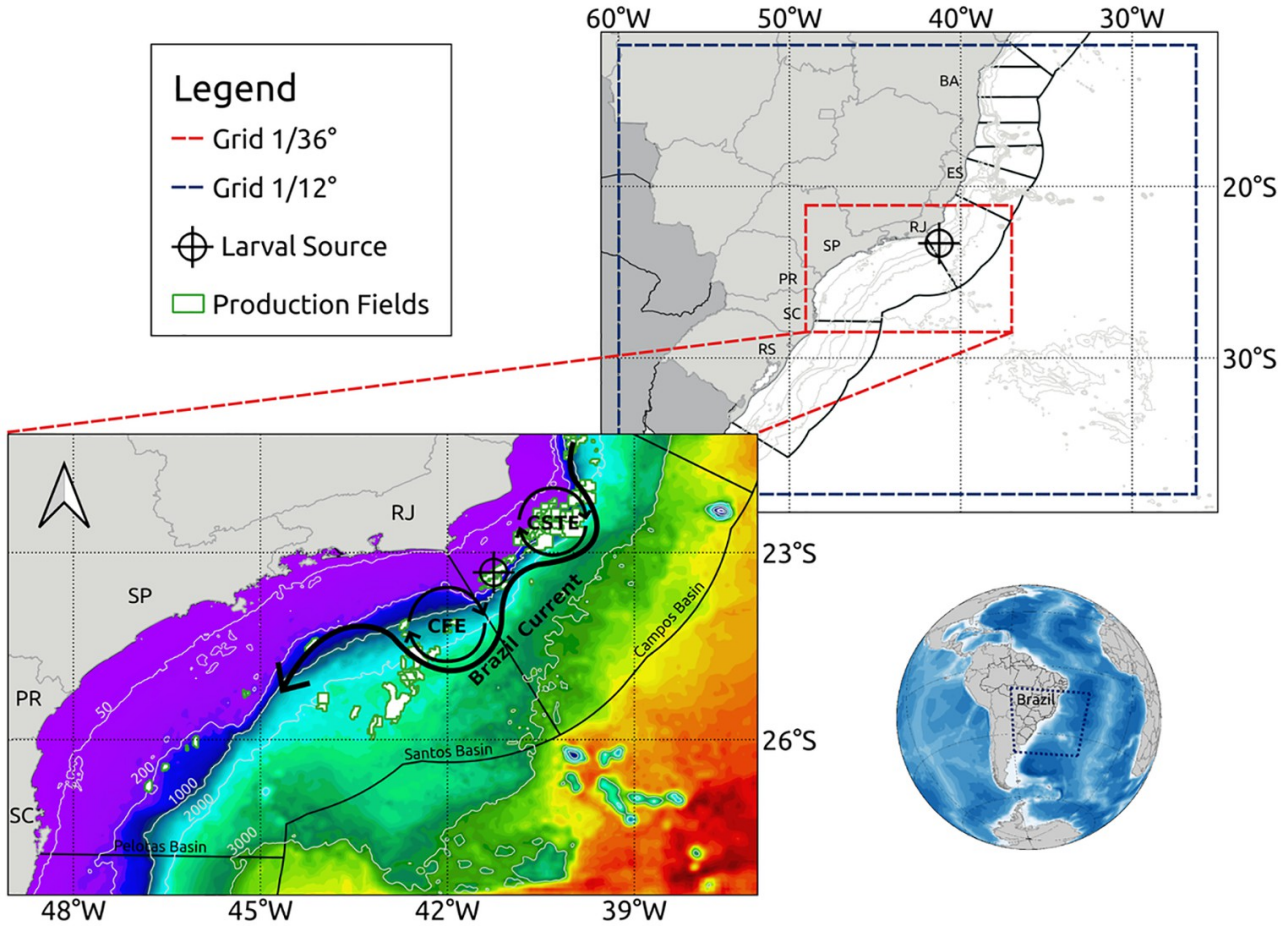
Geographic location of the grids used within the simulation. The blue dashed line represents the coarse grid domain (1/12°), while the red dashed line indicates the nested refined grid domain (1/36°). Below is possible to observe the nested grid in more detail, where oil and gas fields are displayed (white with green borders polygons) among the larval source used for the dispersal simulation (cross-circled marker). Schematic Brazil Current, Cape Frio Eddy (CFE), and Cape São Tomé Eddy (CSTE) primary circulation are also shown. The colors indicate the bathymetry of the nested grid, and the isobaths of 50, 200, 1000, 2000, and 3000 meters were extracted from public NOAA data ETOPO1 [48]. Further, the Campos and Santos basins perimeters are represented by black lines. The following Brazil states are represented: BA (Bahia), ES (Espírito Santo), RJ (Rio de Janeiro), SP (São Paulo), PR (Paraná), SC (Santa Catarina) and RS (Rio Grande do Sul).

Dispersal of biological species responds to large-scale disturbances, which condition population dynamics [1, 2], networks of biological interactions [3, 4] and evolutionary patterns [5–7].Most marine invertebrates evolved maintaining a pelagic phase, being subject to transport by ocean currents, which allows them to escape unfavorable conditions for their development [8, 9]. In general, invasive marine species show broad phenotypic plasticity, with extended planktonic phase and longer dispersal scale [10, 11]. In this sense, as their range increases colonization of new areas is possible [12]. Some mesoscale features, like eddies, can alter the expected distribution pattern of Brazil Current flow. Eddies are known to accumulate and redistribute biomass, such as phytoplankton and zooplankton, not only in the surface but also with a vertical signature [13]. Some studies have already developed mathematical models applied to dispersal simulations of benthic marine organisms, based on larval stage duration data [14, 15]. Sun Corals, *Tubastraea* spp., originally from Pacific and Indian Oceans [16, 17] have invaded natural and artificial areas of North and South Atlantic Oceans e.g. [18–23]. These alien corals were registered for the first time in Brazil in Rio de Janeiro State in the 80s [24]. Since then, they have established stable populations scattered along rocky shores between Bahia (BA) and Santa Catarina (SC) states (location of the States on Fig 1) [25]. Reports published over 20 years point to Sun Coral ability to colonize bare artificial surfaces, such as oil platforms, as the leading cause of the establishment of this specie outside its native distribution area [24, 26–29]. The Sun Coral harm caused in invaded reef systems includes changes in ecosystems populations, displacement of important endemic corals and other sessile eco-engineering species of reef benthic assemblages, compromising ecosystem functions [30–32]. In addition, negative impacts on fish are expected, as the main prey for water column consumers has been described as reduced [23]. Furthermore, the negative impacts may be intensified by the presence of *Tubastraea* spp. through the facilitation of other invasions [33, 34]. Consequently, many marine habitats are in danger, including the world’s largest rhodolite bank and the more extensive and richer biogenic reef of the South Atlantic [35]. The ocean regions of Campos and Santos basins have one of the most productive oil and gas fields in Brazil and the Sun Coral colonies are associated with it. This area (Fig 1) is dominated by the Brazil Current, a western boundary southward current, that flows in the upper 500 m with velocities greater than 0.5 m/s along the continental shelf break and its core centered around the 1000 m isobath [36, 37]. Below this flow, there is an Intermediate Western Boundary Current (IWBC) that, off the southeast Brazilian coast, flows northward with a core around 800 m deep with typical velocities exceeding 0.25 m/s [38, 39]. The Cape Frio 23°S (CF) and Cape São Tomé 22°S (CST) areas have a complex mesoscale activity due to the baroclinic instability of the system, BC—IWBC, that presents an abrupt change in the flow direction, from southward to northward [40, 41] due to the interaction between topography and the ocean currents [42]. In this region there are formations of meanders that, occasionally, close out in rings which can be advected, absorbed or emitted by the BC [42–44]. The Cape Frio Eddy is a cyclonic ring originated by a necked meander and has an unstable quasi-stationary growth phase [40, 45]. Most studies focused on how mesoscale processes influence the primary production, but less has been done to understand how these processes influence higher trophic levels such as zooplankton [46]. Although oil platforms are known dispersal vectors, numerical studies suggest that the strong mesoscale activity in the region prevents the propagules from reaching the shore, especially in the Santos and Campos Basin [47]. Thus, due to the presence of oil rigs (most of them with presence of Sun-Coral) and the intense mesoscale activity, this area is particularly interesting in providing an interaction between the mesoscale features and Sun-Coral larvae. This study aims to understand how these larvae interact with the Cape Frio Eddy and which patterns distinguish the dispersal in a scenario of Brazil’s Current stability in the face of a mesoscale event. Here we investigate both the horizontal dispersal patterns of larvae (aggregation or dispersal), as well as vertical displacement, distances traveled, and regions predominantly occupied by sun coral planulae.

The “The Numerical Model” section of this article is dedicated to explain the hydrodynamic numerical modeling, the eddy tracking algorithm and the coupled dispersal module. “Results and Discussion” section presents and discusses the results of the ocean circulation model and its validation based on mean currents, thermohaline fields, ARGO profiles and the mesoscale eddy characteristics. Also, the larvae experiment results with horizontal and vertical analysis are exposed. “Conclusion” section summarizes the results and provides the final conclusions of this study.

## The numerical model

### The hydrodynamic experiment

**Fig 2:**
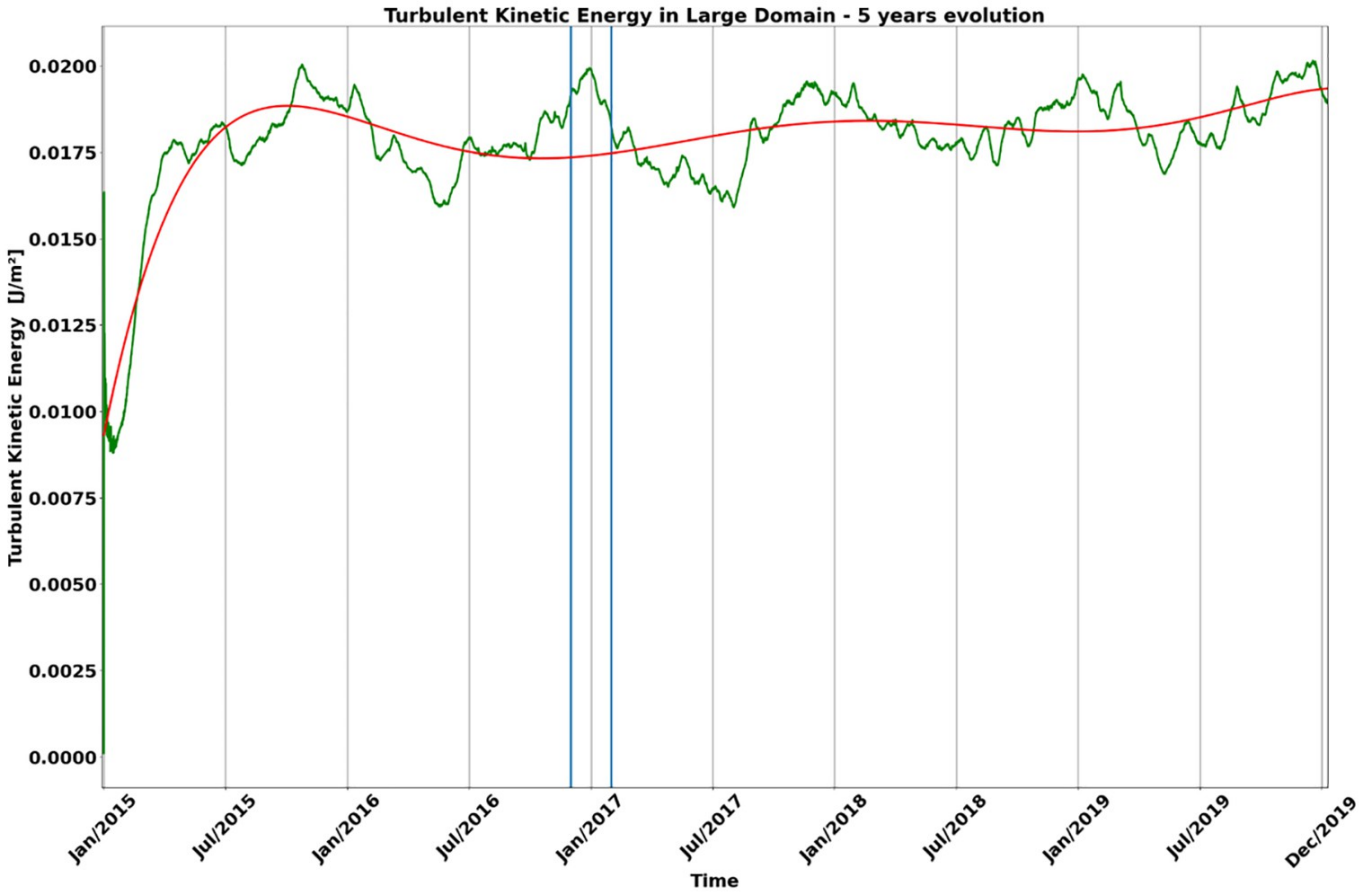
Temporal evolution of turbulent kinetic energy over five years of simulation in the large grid (1/12°) experiment. The red curve is a polynomial adjustment, in which we see the model’s stabilization trend. The two vertical blue lines represent the period of larval dispersal simulation (3 months).

The hydrodynamic model Regional Ocean Modeling Systems (ROMS) [49] was implemented to generate the currents and mass fields which later fed the dispersion experiment. In a first approach, a grid of 1/12° (∼9 km) of horizontal resolution and 32 vertical sigma levels was created from the bathymetric data measurements of the Global Relief Model (ETOPO1), which is a model that integrates topography, bathymetry and coastline data into a single database [48]. There was no smoothing of the bottom topography to make the bathymetry of the region between 63°W to 24°W and 38.3°S to 9.5°S as accurate as possible (Fig 1). This grid was nested within a second high-resolution grid, for southeastern Brazil region, 49°W to 37°W and 28.5°S to 21.1°S, with 1/36° (∼3 km) of resolution and also 32 sigma levels (Fig 1). The second grid, fed by the edges with properties of the larger grid, allows observing the behavior of the features on a suitable scale for the proposed studies of larval dispersal in a quasi-stationary Cape Frio Cyclonic Eddy evolution. The ROMS is a 3D model that solves fluid motion primitive equations with hydrostatic and Boussinesq approximation, using a “terrain-following” vertical coordinate system. The large domain has three open contours (north, south and east) and one closed (west), accompanying the Brazilian coastline. In the open contours it is necessary to establish analytical rules to define the appropriate behaviors of the model variables in their limits. The boundary conditions of radiation and Flather [50] were used to solve, respectively, the baroclinic and barotropic velocities. The implicit Chapman [51] condition was used for the free surface variable. The gradient boundary condition was used for temperature, salinity and turbulent kinetic energy. According to the indicated analytical rules, the boundaries are fed by tri-dimensional properties derived from climatological data [52]. These combinations ensure that the inputs and outputs at the edges align with the typical average ocean currents. While a relaxation to climatology might pose limitations in the context of a forecasting model, it has been suitably tailored for the process model to faithfully replicate coherent mesoscale activities, particularly those characterized by the Brazil Current and its eddies, maintaining a high level of coherence. These implementations enable communication between the model inside and the open edges (https://www.myroms.org/wiki/Boundary_Condition). For the monthly adjustment, there is still a relaxation to climatology inside the grid, to correct the derivation of the mass field during the stabilization run. Thus, the transition between different climatological fields is made of continuous form and without sudden variations, because climatology is applied to suppress its drift and/or bias. Generally applied to water temperature and salinity in monthly cycles, the impact of this adjustment significantly reduces errors in the average and annual cycle of long-term model results. A five-year integration run was carried out from January/2015 to December/2019 in the large grid, aiming to create a stable initial condition for the evolution of the larvae experiment in the nested grid. This simulation was conducted with atmospheric forces dated every 3 hours. These input data are at a regular grid of 0.25° from the European Atmospheric Reanalysis (ERA5), which represents the latest generation of European Centre for Medium-Range Weather Forecasts reanalysis. The experiment used “bulk fluxes” formulations [53] to calculate the air-sea interaction fluxes. Climatology data of the World Ocean Atlas 2009 (WOA) [52], a product composed of *in situ* and interpolated measurements for a grid with 1° of horizontal resolution and 24 vertical levels, was used to compose the thermohaline fields for boundary, initial conditions and nudged climatology. The experiment reached the stability of dynamic properties within 6 months (Fig 2). From this, a typical Cape Frio Eddy scenario was chosen to restart the simulation and release virtual larvae in high-resolution. The nested simulation was performed one-way, maintaining the atmospheric forcing from the larger grid, using the result from third November 2016 as the initial condition of the three-dimensional thermohaline field for a simulation period of three months.

### Eddy properties and metrics

To quantify the capacity of the Cape Frio cyclonic eddy to trap a volume of water and, consequently, planktonic organisms in its interior, was used a nonlinear parameter (*U*/*c*) proposed by [54]. The dimensionless ratio *U*/*c* > 1, where *U* is the maximum circum-average geostrophic speed within the eddy interior (swirling speed) and *c* is the translational speed of the eddies, implies that the *U* velocities are as large as or greater than the translation velocity (*c*). Thus, the eddy can be considered as a nonlinear wave disturbance that propagates through a quasi-stationary scenario, as seen in typical cyclones generated in the BC [39]. According to [54], as this ratio increases, the eddy can trap more water and modify its regional properties through advection of a trapped fluid parcel. The advection of these waters implies that eddies can transport water properties such as heat and salt, affecting the momentum and heat fluxes, as well as nutrients, phytoplankton and zooplankton flow, thus having considerable effects on the dynamic of marine ecosystems. Given that unstable cyclones are frequent in BC [40, 44, 55], and considering fluid entrapment processes is essential to detect the potential capture of sun coral larvae by nonlinear cyclonic eddies in the study area, it’s extremely important that the simulated CFE represent correctly this characteristic. In the tropics, where *U*/*c* tends to be lower, about 90% of the combined cyclonic and anticyclonic mesoscale features had *U*/*c* > 1. However, tropical eddies are strongly nonlinear; In the extratropical zone, 48% have *U*/*c* > 5 and 21% have *U*/*c* > 10 [54]. In the case of the BC cyclones, values above 5 are expected, as seen in [41]. From the daily results of the hydrodynamic model, eddy properties were obtained using the py-eddy-tracker code [56]. This code uses an sea surface height (SSH) approach loosely based on the methods described by [57, 58], providing identification of variables such as amplitude, effective radius, distance (translation speed) and swirling speed. For identification, the code uses closed contour analysis, corresponding approximately to the streamlines of geostrophic flow, that must meet a series of criteria related to shape errors, pixel count, amplitude threshold, and no more than one maximum or minimum within the interior. There is a possible limitation for identification of highly asymmetric eddies with more than one maximum or minimum as well as in regions near the equator due to the non-geostrophic balance. Based on the centroid position obtained in the identification stage, the tracking process is carried out according to a set of dimensionless similarity parameters influenced by distance, area and amplitude [56]. The py-eddy-track algorithm was used to access the Cape Frio cyclonic eddy properties along the first 30 days of larvae analysis. Variables provided by this method, such as rotation and translation speed were used for accessing the nonlinear metric exposed by [54].

### The dispersion experiment

One of the oldest methods of ocean circulation study is the release of lagrangian derivatives [59]. Here a native subroutine of ROMS (Floats) coupled to the hydrodynamic results on the nested grid was used to address the dispersal patterns. This module considers particles released at specific predefined settings and depths, which are derived in a lagrangian way, evolving with the field of adjacent currents. Also, the vertical diffusion is represented by a random walk technique, providing solutions of vertical displacement within a different timescale, allowing a better representation of the vertical distribution of the planulae. This technique is commonly used to model environment diffusion, and it is incorporated in ROMS subroutines for vertical mixing based on a random walk [60] that work together to prevent spurious mixing and vertical velocity nudging. The module represents, in a very preliminary way, the basic characteristics of *Tubastraea* spp. larvae, such as the depth at which the larvae are transported, that according to [61, 62] is restricted at the superficial level. On the other hand, they are lecithotrophic larvae, which do not feed and have reduced mobility [63–65], practically null for the present metric scale. This approach allows us to isolate the role of physics (e.g., current strength and direction) on dispersion in the region (e.g. [28, 66, 67]). For the release of larvae, the Campos basin, where the most important oil platforms are located, was chosen as a source spot with a cluster of three points centered at 41.255°W, 23.316°S (larval source in Fig 1). This spot is in a highly dynamic place, where the Brazilian coastline changes direction, from predominant north-south to east-west orientation, implying particular mesoscale features like meanders and eddies. The simulation released 3000 larvae per day during a planulation event of 16 days in November, emitting an amount of 48,000 larvae during the entire period of simulation. This release period was chosen due to the presence of a cyclonic eddy and because, among other times, November is a reproductive activity season of Sun Coral for the CF region [68]. These quantities of larvae were considered a good approach to investigate their dispersion in an eddy event. The competence window period in which the larvae retain the capabilities to settle and perform metamorphosis is a key factor affecting the dispersal potential of benthic organisms that disperse via lecithotrophic larvae [63, 64, 69]. Although most sun coral larvae released in laboratory experiments settled in a day or less, these planulae perform metamorphosis even after floating for 40 days in the water column, and are capable of surviving for periods of up to three months so it has a high dispersive potential and can be transported by ocean currents for long periods [65, 70].

## Results and discussion

### Validation of the hydrodynamic model

**Fig 3:**
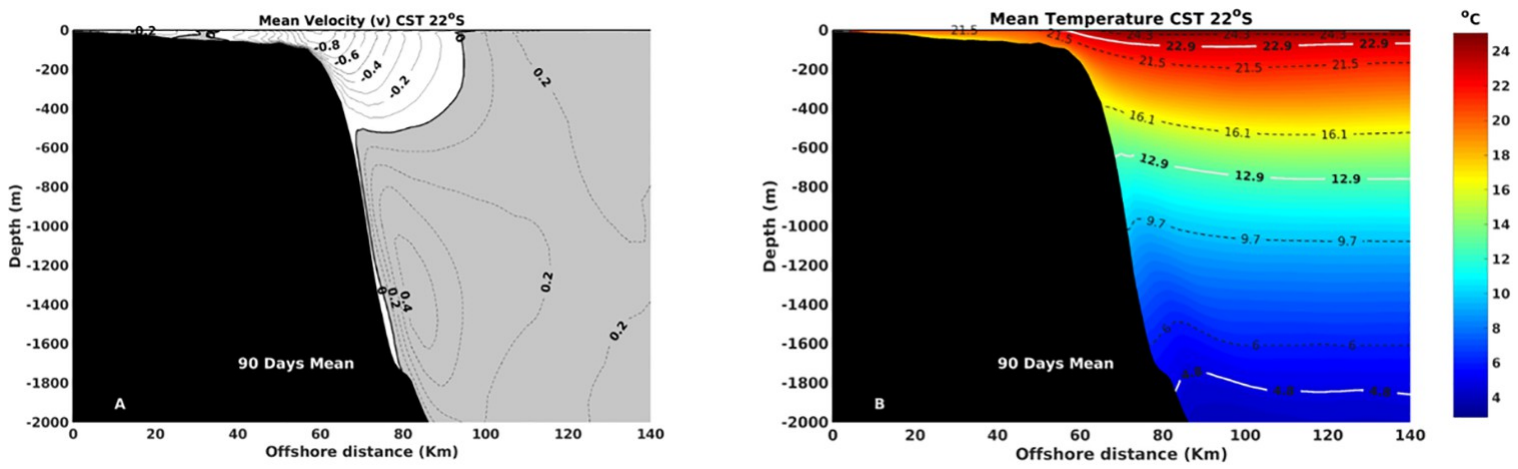
On the left (Fig 3a), the mean zonal section in Cape São Tomé shows the vertical distribution of meridional velocity during the nested experiment period (Nov/Dec/Jan). The continuous contour lines are southward velocities, with a thicker one representing the level of no movement. Dashed contours are northward velocities. On the right (Fig 3b), the lines are isotherms displaying the vertical temperature distribution. Warm colors represent high temperatures, while cool colors are the lower ones. The white isotherms are the mean value of the Tropical Water, South Atlantic Central Water and Antarctic Intermediate Water, from surface to bottom, while dashed isotherms are the standard deviations [40]. The black area represents the continental shelf.

Ocean models operating without data assimilation may not fully capture all aspects of ocean variability. However, for the purposes of this study, achieving a reasonably accurate representation of typical mesoscale oceanic variability is entirely sufficient. Given the specific focus on variability, the primary priority lies in ensuring the accurate depiction of the emission cycles of the BC eddies, which is inherently linked to its mesoscale characteristics. Furthermore, it is crucial to accurately portray the depth structure of the BC for this research, because the interaction between BC with IWBC forms a strength baroclinic system, responsible for the quasi-stationary growth of the CFE. Therefore, comparisons with *in situ* vertical profiles, climatological data, and flow structures inferred from the existing literature are shown. The model results were set to be stable and reproduce typical large-scale circulation and mesoscale scenarios of the Western South Atlantic, as the Brazil Current flow, its meanders, eddies and the interaction between BC and Intermediate Western Boundary Current. More specifically, the simulation implemented on ROMS could establish a precise position for the BC, flowing in the first 500 m along the shelf break, and estimate the directions and intensities of the velocity fields, as illustrated in Fig 3a. The zonal section was chosen on account of the proximity to the site where the larvae are released and to provide comparison between previous studies [39, 71, 72]. The level of inversion in currents flow is a crucial feature for the baroclinic instability and this is accountable, as stated by [40], for the BC quasi-stationary eddies growth in the SE region. Therefore, a reliable representation of the inversion depth is essential for creating an eddy resolving model for this region and further simulating an event of larvae dispersion in one of these eddies. The results, presented as a thicker line of zero velocity between the two main fluxes around 500 m in Fig 3a, show conformity with [72], which referred to 450 m as the inversion level. Beneath this, the IWBC was also represented in position, with its core around 1000 m and northward velocity direction. These characteristics, for both current systems, are in good agreement with [39, 40, 72], showing velocities exceeding 0.5 m/s located in the upper 500 m and, for IWBC, northward fluxes surpass 0.3 m/s around 1000 m in SE regions. The same pattern can be seen in Fig 3a, in which ROMS velocity is presented in a mean section located in CST during the larvae dispersal analysis. It is possible to observe the BC in the white part of the figure, flowing southward with its core represented by 0.8 m/s in the upper 500 m. The IBWC is expressed by a core of northward velocities up to 0.4 m/s around 1000 m.

The vertical mean temporal temperature section in the upper layers is characterized by warm waters offshore on the superficial level (Fig 3b). On the other hand, on the continental shelf, the waters are colder if compared in the same depth on the offshore region, where BC is predominant. This temperature difference is possibly due to the upwelling of cold, nutrient-rich, deeper waters in the coastal region of CST [55, 73]. In this region, the BC carries Tropical Water (TW) and South Atlantic Central Water (SACW), with mean temperature values of 22.9°C and 12.9°C, respectively [36, 40].

**Fig 4:**
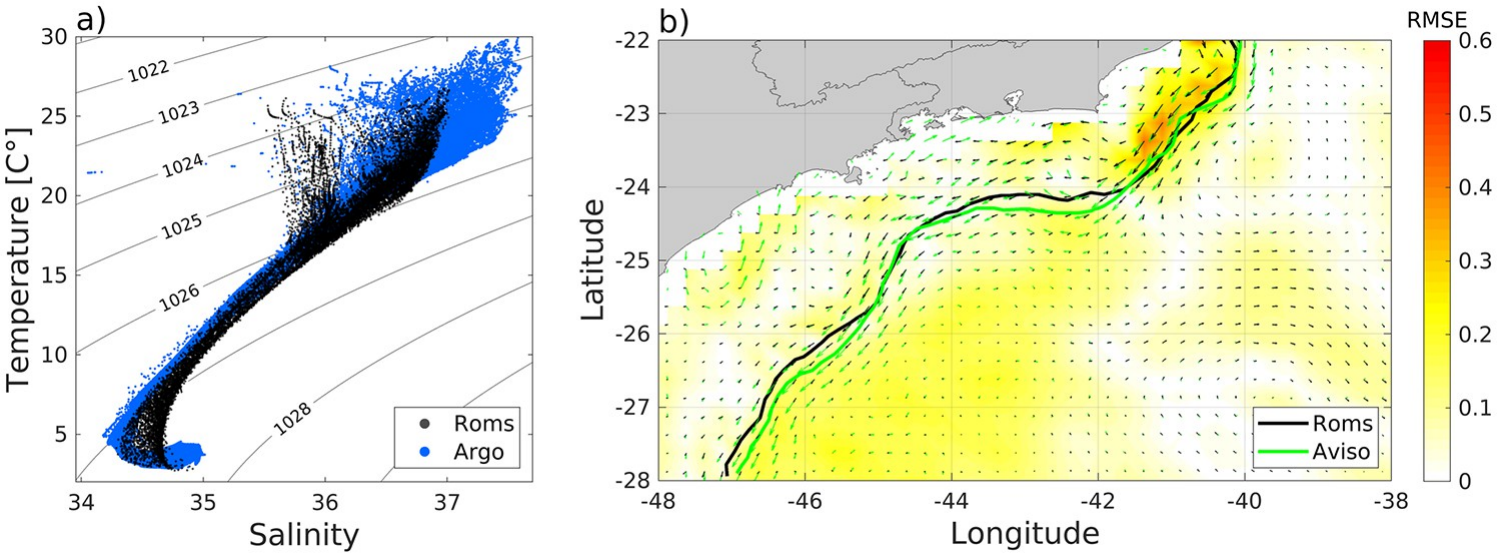
In this panel (a), a comparative T-S diagram is presented, featuring data points from ROMS indicated by black markers and those from ARGO profilers by blue markers, spanning the years from January 2015 to December 2019. Painel (b) illustrates the average currents for the same period, sourced from (www.aviso.altimetry.fr) in green, alongside corresponding average current data from ROMS in black. The lines are representative of the maximum velocities axis for the Brazil Current within AVISO dataset and ROMS. Direction and intensity vectors are also displayed in matching colors. In the background, the Root Mean Square Error (RMSE) in meters per second highlights the discrepancy between the two datasets.

Data obtained from the ARGO profiler’s Drifters project [74] played a fundamental role in validating the model’s implementation between 2015 and 2019. This validation process involved an examination of temperature and salinity data extracted from *in situ* profiles of ARGO drifters, which were inside 22°S-28°S. This comparative analysis has enabled us to assess the model’s capacity to accurately replicate the stratification observed within the study area. A set of 686630 individual observations is displayed against seasonal averages for each simulated year (2015-2019), as demonstrated by the T-S diagram (Fig 4a), where it becomes evident the alignment of the primary oceanic water masses [36]. Even though the model exhibits a slightly cooler and less saline surface layer, there is, in general, a satisfactory representation of the thermohaline structure.

To expand the scope of the validation, Fig 4b shows a comparison between the model’s output and average geostrophic velocity data from the Unification and Altimeter Combination System (DUACS), released by AVISO (www.aviso.altimetry.fr), covering the period from January 2015 to December 2019. The figure illustrates the Root Mean Square Error (RMSE) between the mean measured data and the mean ROMS velocities. The maximum RMSE value is approximately 0.5 m/s, occurring on the shelf break of Campos Basin, a region of high variability of the BC, with meanders and eddies. Further, to verify how accurately the model reproduces the average position of BC’s main axis in comparison to *in situ* data, the axis of maximum velocities from the dataset was digitized. This comparison, visually represented in Fig 4b, demonstrates a satisfactory alignment between the model (black line) and observations from AVISO (green line). The results adequately reproduce the currents in the region, a crucial factor for dispersion studies, thus underscoring their reliability for further research endeavors.

**Fig 5:**
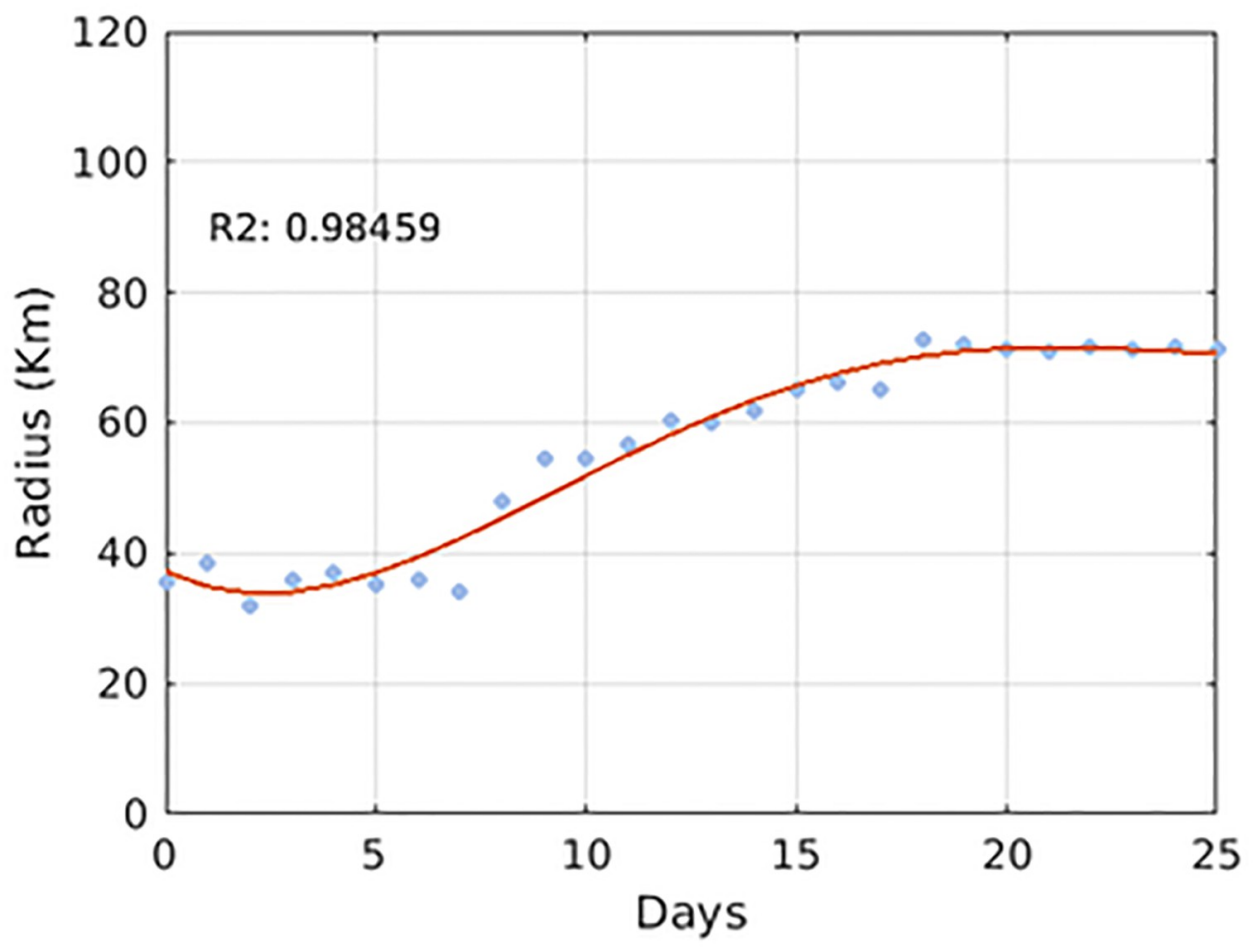
Temporal evolution of the CFE radius with mean growth rate value of 0.052 day^−1^. The red line was fitted with a good approximation of 0.98 (r-square), allowing easier observation of the growth stabilization around day 15.

**Fig 6:**
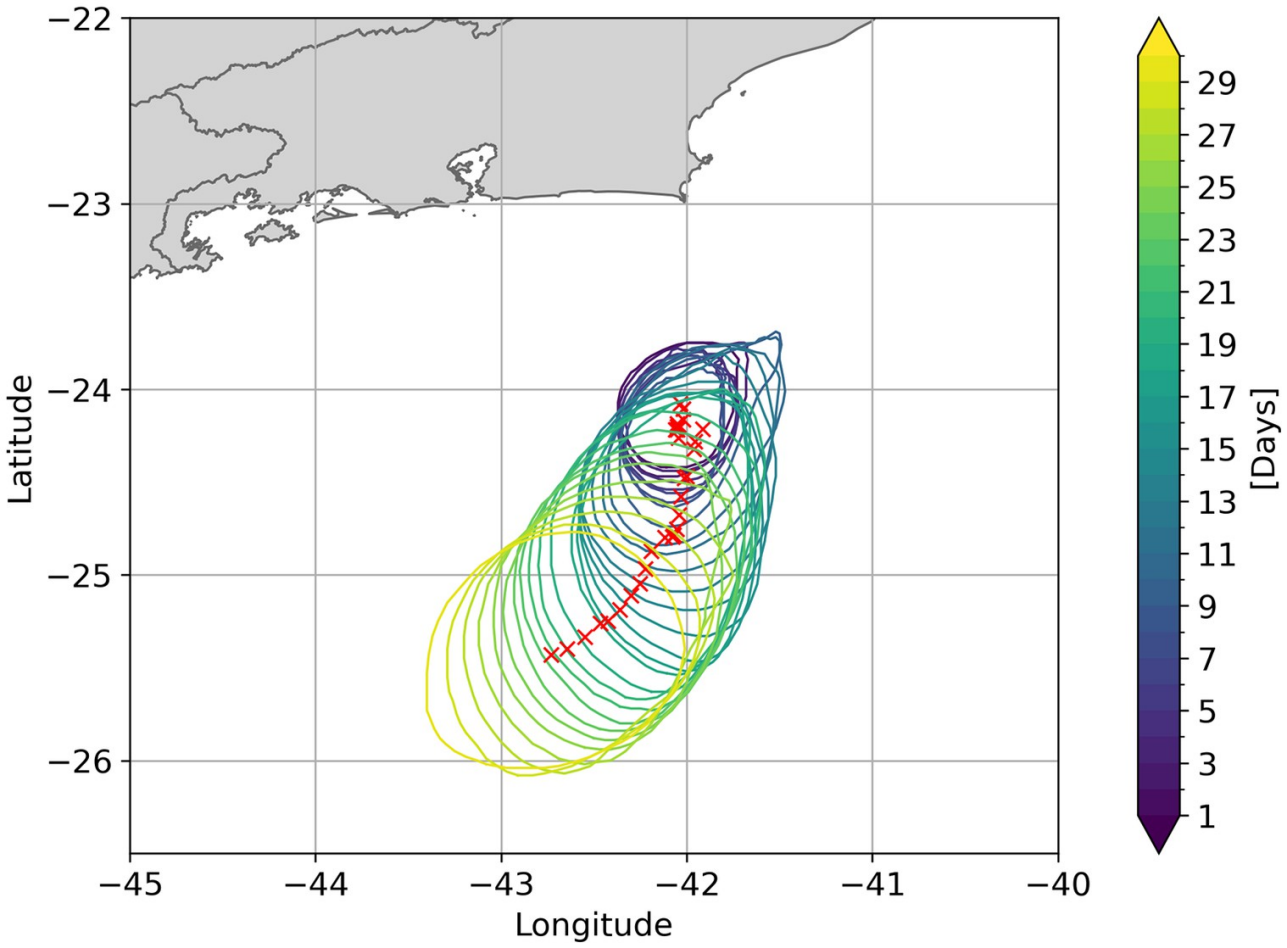
Tracking of the Cape Frio cyclonic eddy event, showing growth and migration patterns over 30 days obtained by the py-eddy-tracker code [56]. The red X represents the eddy’s core as it evolves. Eddy contours are also represented with colors varying regarding temporal evolution. Cool colors indicate the first days of tracking while the warm ones, the last days. Amplitude, effective radius, distance (translation speed) and swirling speed were accessed from this method.

It is also necessary to evaluate typical eddy characteristics. In general, extratropical eddies are highly nonlinear, with 48% having *U*/*c* > 5 and 21% having *U*/*c* > 10. According to [54] these values are found in regions of highly meander currents, mainly in the western boundary ones, such as the Brazil Current. For the Southeast region of Brazil, this reference indicates values of this metric around 9 and the present study found a value (10.6 ± 6.1), which classifies the CFE as highly nonlinear (Table 1). Another work that analyzed the *U*/*C* parameter for this eddy was conducted by [41], which achieved a value of 7.5. As it is nonlinear, Cape Frio Eddy can trap not only water in its interior but, heat, salt, phytoplankton and zooplankton that can also be transported within this feature. Therefore, sun coral larvae may remain trapped within this mesoscale feature. The CFE presents a quasi-stationary growth, with growth rates varying from 0.06 *day*^−1^ to 0.3 *day*^−1^ showing mean radius values of 57 (± 14) km and 63 km [40, 41, 72]. Using the py-eddy-track algorithm that identifies and tracks eddies from sea surface height fields, values of 0.052 *day*^−1^ growth rate were found, with a growth stabilization around the 15th day and a mean radius of 51.9 (± 14.8) Km. Also, [54] shows the region of CFE with a mean eddy scale of around 70 km. Based on the comparison between long-term mean metrics present in the above references the results represent, in a realistic way, the CFE (Figs 5 and 6).

**Table 1:** Statistics metrics of the CFE during 30 days of tracking.

Parameter	Mean	*σ*	Min.	Max.
Radius [Km]	57.6	14.9	31.9	72.6
Translational speed (c) [m/s]	0.081	0.040	0.013	0.172
Rotational speed (U) [m/s]	0.67	0.17	0.40	0.94
U/c	10.6	6.1	3.5	30.2

In the first 15 days of larval experiment, the CFE growth is quasi-stationary and as it increases it is advected southward by the mean water flow due to the Brazil Current. As mentioned by [75], the offshore displacement of the CFE is also intricately intertwined with complex interactions involving other eddies formed within the region, as well as those that arrived in the region. This pattern is illustrated in Fig 6, where a high density of red markers at 24.2°S and 42°W represents the initial growth in a stationary phase. From the 15th day forward, it starts a more prominent translation pattern shown by the yellow tones in bigger circumferences and the greater distances between centers. While this translation is taking place, the larvae are subject to the eddy dynamics, generating, for example, a more restricted distribution than those outside the eddy. Furthermore, vertical velocities can be intensified by the eddy field [76], so the larvae are subject to a vertical upward/downward displacement as an upwelling/downwelling occurs.

In order to substantiate the circular flow patterns generated by the presence of eddies in the area, it is possible to conduct a comparative analysis with trajectories of Surface Velocity Program (SVP) drifters tracked via satellite, as delineated by [77]. The research referenced herein employed data derived from *in situ* Langrengean drifters, which effectively elucidated the impact of enduring mesoscale structures within the Cabo Frio region on the behavior of the Brazil Current.

### Larvae dispersal

**Fig 7:**
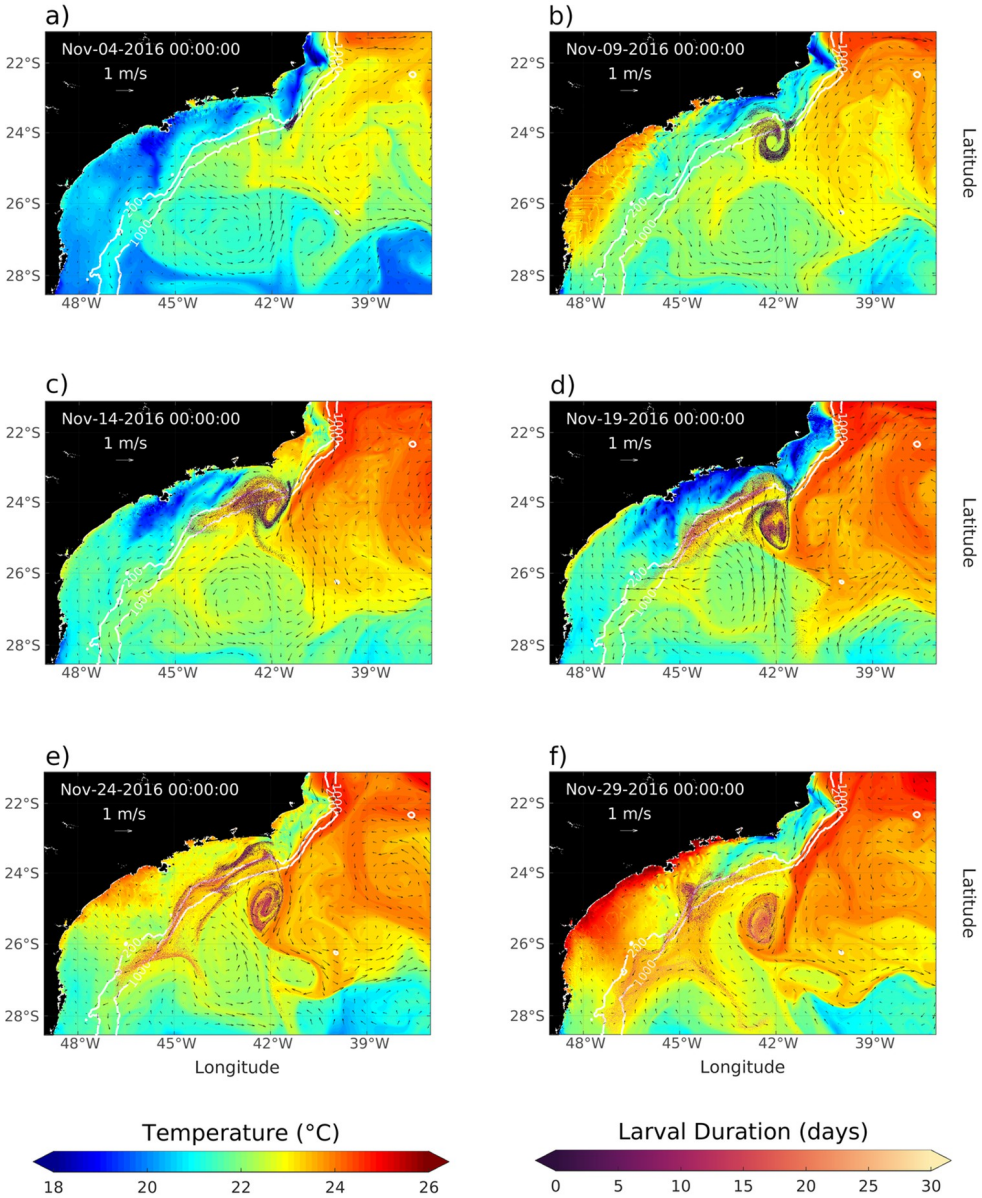
Larval dispersion during 25 days of simulation displayed on a 5-day time step. The figures are illustrating the hydrodynamic field (arrows) among sea surface temperature (colors in the background) for Nov-04(a), Nov-09(b), Nov-14(c), Nov-19(d), Nov-24(e), and Nov-29(f). Furthermore, larvae are represented by colored markers depending on larval age. The clearer tones indicate older larvae and the darker tones are younger. The 200 and 1000 m isobaths are also shown in white strokes.

On Nov 4th, after 1 day running, an initial condition of the dispersion event is presented, with larvae near the released location but beginning to disperse in a Southward offshore direction also showing sea surface temperature in the background colors (Fig 7a). The currents field shows a meander off Cape Frio, which evolves and pinch off in an eddy. Five days later, the larvae sensed the eddy presence, causing a clockwise loop on them. Also, a branch gets close to the 100 m isobath, and another eddy region feeds with young larvae (Fig 7b). On Nov 14th, it is possible to distinguish two patterns: one core of larvae going southward along the shelf break and another mass in the eddy vicinity. In this last region, both young and old larvae are noted (Fig 7d), represented by the darkened and lightened colors, respectively. In most extern boundaries of the eddy, young larvae are dominant, and as the focus moves toward the center, the older ones are more present. The larvae advected by the Brazil Current moves away from Campos Basin, crossing, on Nov 19th, the 26°S parallel. About 70% of total larvae released are free, not trapped in the CFE, and were also advected south-southeastward to the middle of the domain region. Although 30% of larvae are trapped in the eddy at this time, the space occupied by these is much smaller than the free amount scattered along the domain, so the presence of the eddy is significant to concentrate the larvae. On Fig 7f, the eddy is detached from the BC and still contains planulae inside, 20%. Although they were a minority and in the subsurface, it is noted the presence of 9% of larvae in waters with temperatures below 20°C, which can be stressful or have a lethargic metabolism, thus the potential of colonization is diminished [22, 78]. A significant result is that no larvae released from the location in Campos Basin reached the coast. Hence, it is plausible to demonstrate that the Brazil Current, with its persistent mesoscale activity, serves as a dynamic barrier, as revealed in [77] study and suggested by [47]. This barrier effectively impedes the influx of planktonic organisms from offshore regions into coastal areas, specifically across the 200 meters isobath. So according to modeled scenario showed here and studies referenced below, the oil platforms in Campos and Santos Basin are not a probable direct vector for the coastal colonization of *Tubastraea* spp., thus other fonts, like biofouling on ships that traffic between coast and platforms, may be responsible for this spread out [47, 79, 80].

**Fig 8:**
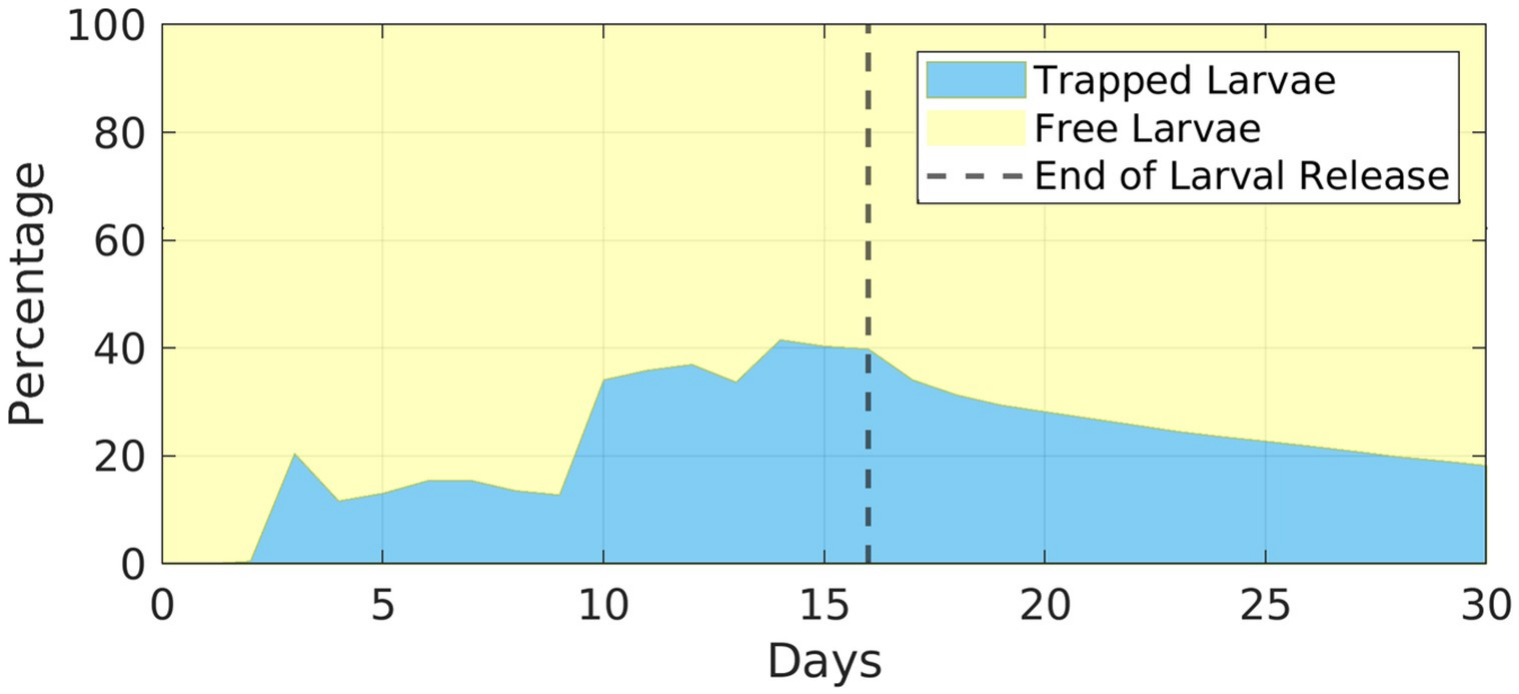
Percentage relationship between larvae inside and outside the eddy during the 30 analyzed days. The vertical dashed line represents the end of larvae release.

Considering the entire domain where the nested experiment was performed, there are two different patterns of larval dispersal. From those who were advected by the mean flux of Brazil Current, a maximum traveled distance of up to 800 km in 30 days was reached. At this final time, about 80% of larvae were outside the Cape Frio Eddy, Fig 7f. The eddy identification method calculates its outer contour, so it is possible to check how many larvae are captured in this mesoscale feature. On the first day of the simulation, there was no planulae captured by the Cape Frio eddy, but from day 2, are observed a sharp increase in this quantity, reaching, from day 10, about 35% of total larvae on the water at that time inside of the eddy. From the total larvae that were released from Campos Basin, the blue area in Fig 8 represents the ones that were trapped in this highly nonlinear mesoscale feature. From day 16 onwards, no more larvae were released (dashed vertical line in Fig 8), so the number of trapped organisms is expected to decrease. After this date, a gradual decrease in the percentage of larvae inside the eddy occurs, reaching 19% at the end of the analyzed period (Fig 8).

**Fig 9:**
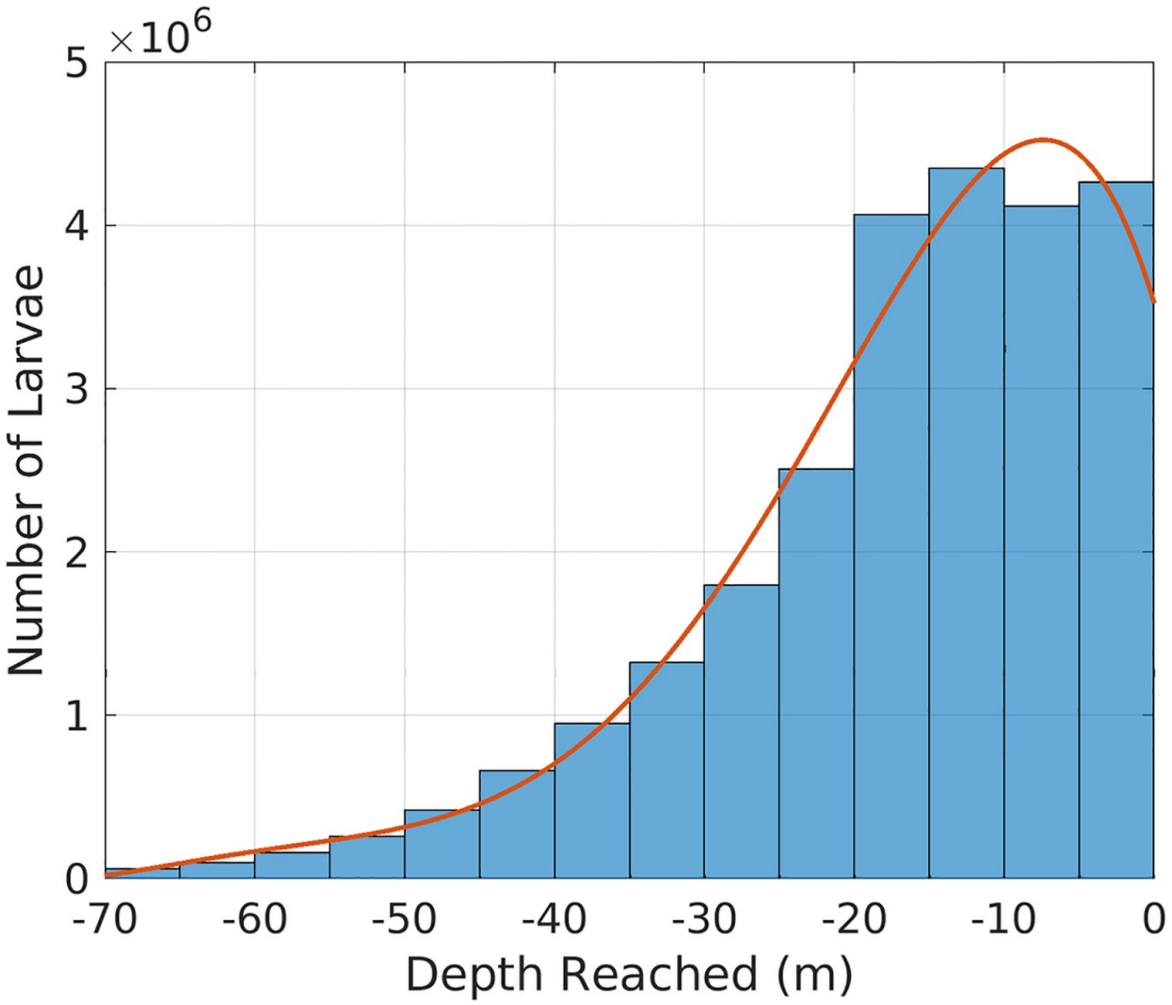
Histogram of larvae distribution with depth, considering all timesteps of each larvae during 1 month of simulation.

Although the initial depth of release is the surface level and most larvae results are still in the first 20 m deep, 1% of total larvae observations reach greater depths of more than 60 m during the first month of simulation, Fig 9. Considering the vertical fluxes induced by eddies migration [46], there are strong indications that the CFE is responsible for this larval sinking (downwelling movement) since these larvae do not perform vertical migrations at this spatial scale.

**Fig 10:**
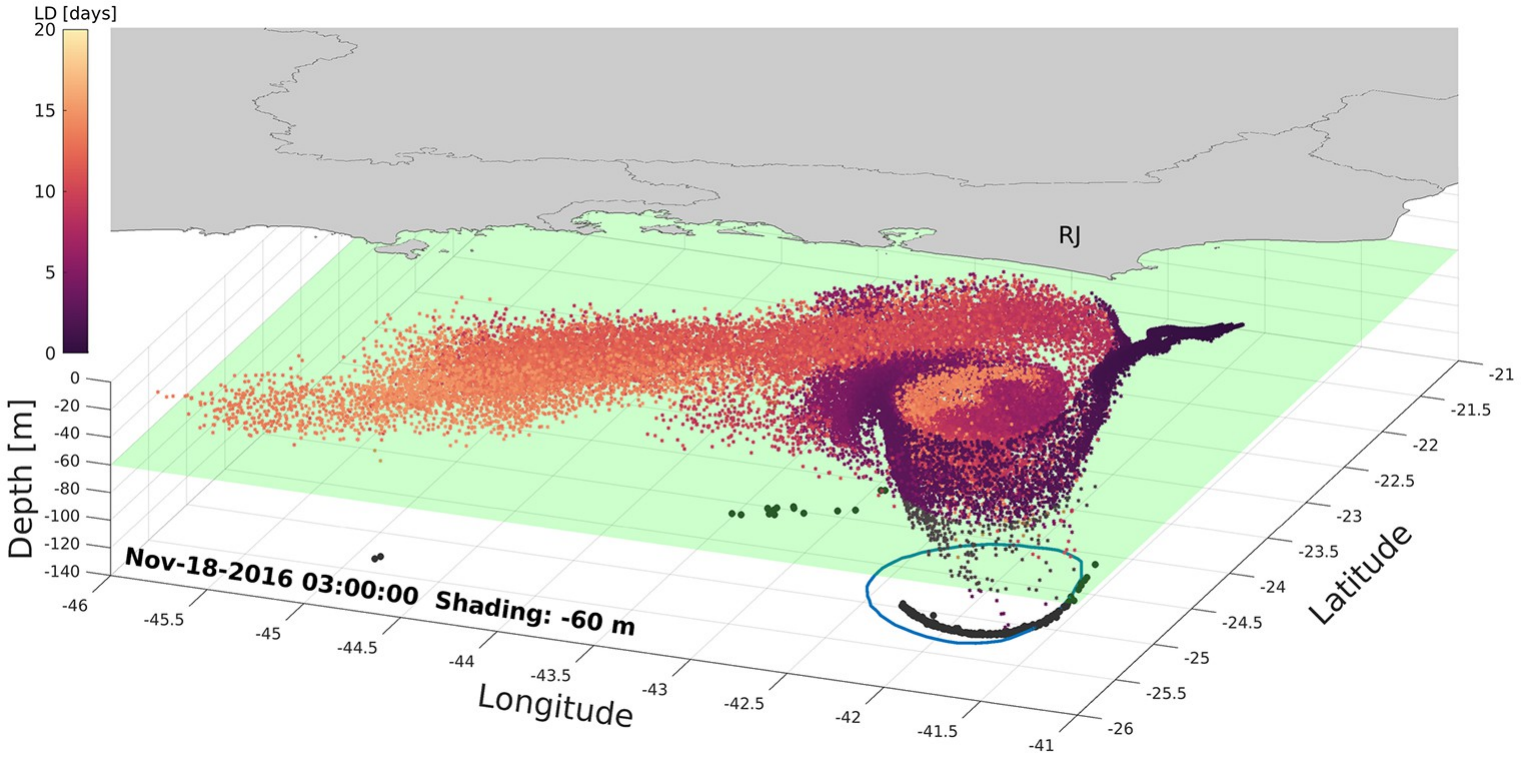
Total larvae released until Nov-18-2016 in a three dimensional view. The colors of the markers represent larvae age, being the clearest ones older, and the darkest ones younger. The green surface is the 60 m level and the blue circle is the CFE outermost contour at this moment. The gray polygon represent the mainland. Also, on the bottom plane, the shadow of larvae that are bellow the 60 m surface is displayed.

Further analysis was conducted to investigate the vertical larval spreading during the simulation period, focusing on understanding the eddy’s influence on larvae depth. November 18 was chosen to illustrate this relationship, where 94% of larvae that are below 60 m are in the eddy vicinity (Fig 10). It is also possible to observe that these deeper larvae are located mainly on the edges of the eddy, indicating the presence of specific dynamics in this region capable of influencing the vertical velocity. Therefore, it is suggested that the larval sinking occurs essentially when they are in an eddy domain. With these analyses it was possible to determine that most larvae that reach deeper levels are associated with CFE and those who do not interact with this feature, stay predominantly on the surface.

**Fig 11:**
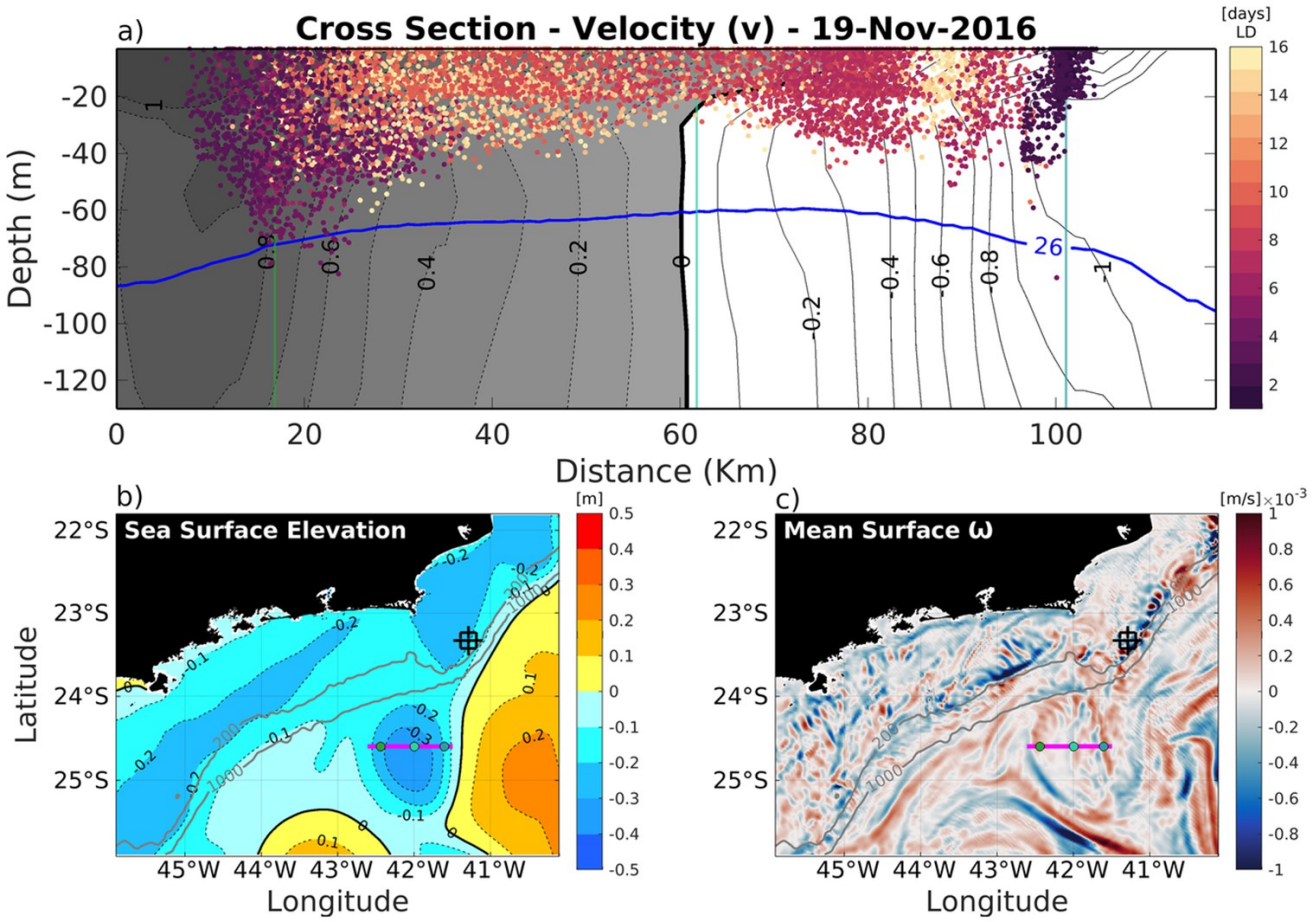
a) cross-section velocity of the Cape Frio quasi-stationary cyclonic eddy, showing the trapped *Tubastraea* spp. larvae, for 11/19/2016 (16 days of simulation). The colors of each marker indicate larvae age since released, so that the white ones are the oldest and the dark ones are the youngest. The blue line is the seawater density 26 kg/m³. b) the sea surface height is represented, with blue colors indicating a depression on the water, and the red ones, a bulge. The levels of the same height are contoured with dashed lines, and the ticker line marks the zero. c) the vertical velocity is presented where blue indicates the upwelling and red the downwelling. The magenta line is the section location, and the three green dots are for better localization as they are presented in (a) as the vertical lines. The gray lines represent the 200 and 1000 m isobaths.

Fig 11 shows a moment on Nov 19th in a vertical cross eddy section plot to investigate the vertical distribution of the larvae in the water column. This date was selected because the CFE was in a representative moment where its shape and amplitude were highlighted (lower left panel), and there was around 30% of total planulae inside the eddy (Fig 7d). A cyclonic eddy has a negative sea surface height, which can be seen by the blue colors indicating a depression in the water at its center, Fig 11b. There are two fluxes perpendicular to this section. On the western side it is northward, shown by the black shade, and southward at the easternmost portion, represented by the white contours, as illustrated in Fig 11a. Both cores are 1 m/s and the eddy center, represented by a black thicker line, is in the middle of this figure (zero velocity). About 80% of trapped larvae are on the upper 40 meters and only a few (9%) are below 60 meters. Analyzing the horizontal distribution of larvae inside the eddy at a fixed depth level, a greater concentration of older larvae (with more than 15 days) can be seen in the middle. In contrast, on the boundaries, younger larvae of less than 5 days are found, suggesting that the dynamics of stationary nonlinear eddy work to aggregate larvae along the time in its center. Also, due to the characteristic upwelling in its nucleus, the cyclonic eddy tends to have a divergent surface flow pattern that will act to thin out the distributions of planktonic organisms in the center [46, 76]. This pattern is shown in Fig 11a. The eddy propagation movements create areas of downwelling (backward) and upwelling (forward) along the boundaries [73]. Although the cyclonic eddies are well known for promoting an upwelling in their center due to the Ekman Pumping, convergence and downwelling often occur in the edges [46]. In this context, there was an investigation on vertical velocity fields to understand the distribution of larvae with depth and wherein the eddy domain it is occuring. The current results have shown that young larvae of about 4 days old can reach depths of 80 m (Fig 11a) in a downwelling eddy area (Fig 11c). On the other hand, on the most right vertical green marker of the section, it is observed that larvae are aggregated in the first 20 m, but at the same point there is also a region of downwelling (Fig 11c). A possible explanation for this ambiguity is that this larvae mass is about 2 days old and near the origin site, where they were released on the surface. In these two days in plankton, the vertical fields on the boundary where these larvae passed by indicated an upwelling event (Nov 17th and 18th), so these factors corroborate the surface concentrations observed in this part of the eddy. In fact, vertical velocities are in a much lower scale when compared to the horizontal ones. Therefore, although instantaneous vertical velocity shows a specific pattern of larvae movement, the analysis must consider under what previous conditions these organisms were subjected. Thus, the hydrodynamic field can act to distribute or concentrate *Tubastraea* spp. in both horizontal and vertical ways and it is worth to mention that even though larvae were released from the surface and have low mobility, they can reach greater depths while interacting with the Cape Frio Eddy dynamics.

**Fig 12:**
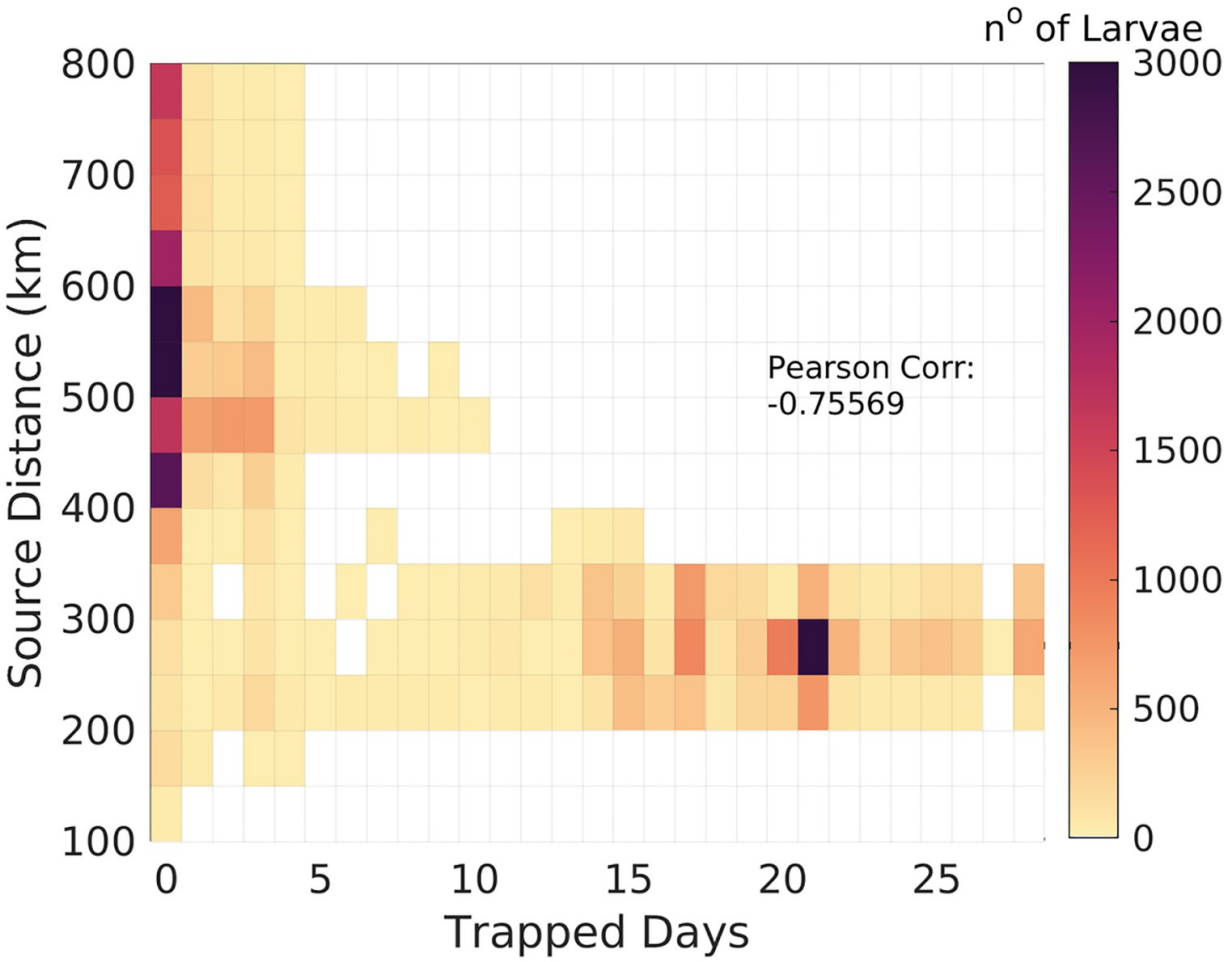
Days inside eddy vs. distance from the origin. The colors represent the number of larvae present in each combined point. Pearson correlation between the distance covered and the time the larvae spend inside the eddy shows a value of -0.75.

Fig 12 is designed to investigate the dynamics of larvae in relation to the presence of the simulated CFE. The calculation of distances is conducted between the larvae’s initial locations, denoting the source point, and their positions at the conclusion of a 30-day experimental period, referred to as the final instant. The outer boundary of the eddy is determined through the utilization of the ‘pytrack’ code, which processes daily sea surface height (SSH) data derived from the simulation. This code serves to delineate the spatial extent of the eddy, a critical aspect of the investigation. At those days, larvae found within the confines of the eddy contour are categorized as ‘captured’ by this mesoscale feature. For each larvae, the days inside the eddy are summed and displayed against the source distance. The relationship between the calculated variables provides comprehensive exploration into the manner in which the CFE influences the trajectory and distribution of larvae over a defined 30-day interval. The maximum number of larvae shown in darker colors represent the two main paths of dispersal. There are 4937 particles that stay 21 days inside the eddy and travel shorter distances (250 km from the released site). On the other hand, a second maximum is shown where 4592 larvae which did not interact with the eddy travel distances of up to 600 km. At the extreme of maximum dispersion, some larvae reached, on day 30 of simulation, 800 km from Campos Basin’s beginning site. The results reveal a strongly negative Pearson’s correlation index of -0.75 between larvae trapped days and source distance, elucidating the quasi-stationary growth rate and the low phase propagation characteristic of the CFE. In this sense, the eddy tends to maintain larvae within its influence domain, acting to reduce the *Tubastraea spp.* dispersal range, as the larvae are transported with it. It is important to note that 9% of the larvae are advected out of the simulation domain and the distance considered for these is of the last position recorded.

According to [40], a possible migration of CFE, mainly to the southwest is a consequence of the BC flow. Eddies can significantly affect the transport of assemblages of planktonic communities as they move away from the formation site, due to the capture of the seawater that contains them. Larvae simulation obtained for this research results in a typical value of 250 km as the migration distance for CFE, Fig 12. Correlated with this, applying the Python track algorithm, a value of 177.7 km was obtained for the maximum distance traveled by the same eddy. [41] reported 180 (± 201) km as the mean traveled distance for CFE. Thus, these different approaches lead to close values for this variable and help confirm the predictions of larval transport as a tracked metric for the Cape Frio Eddy.

**Fig 13:**
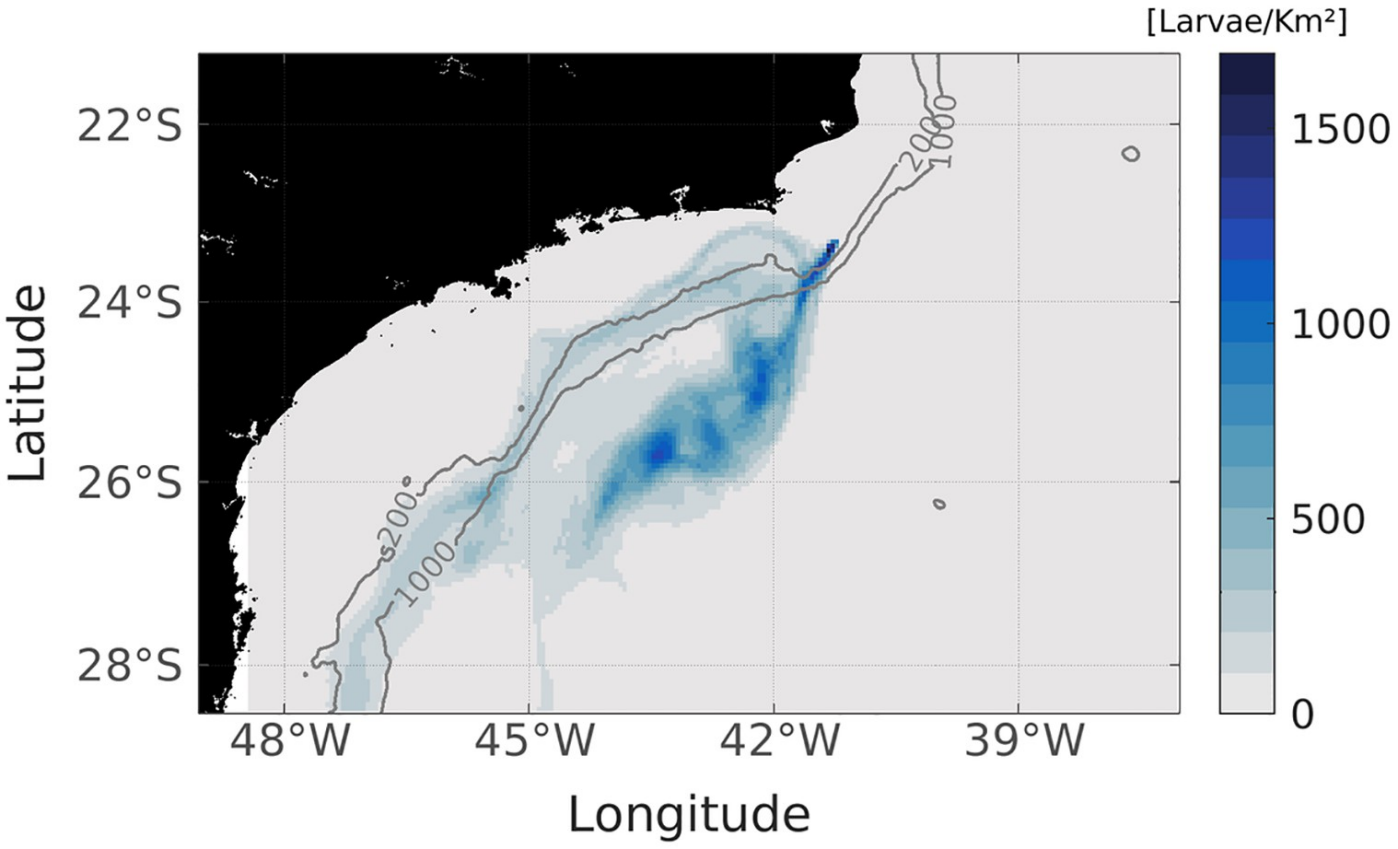
Points where there were larvae present. Stronger tones indicate high larval density along the 30 days, while fewer larvae occur in transparent areas.

Relatively few studies have been done focusing on an approach to how *Tubastraea* spp. larvae are dispersed by the geostrophic currents, but [80], suggested using a lagrangian model, that oil and gas platforms offshore in Santa Catarina State are not a source of larvae for Arvoredo Marine Biological Reserve. However, the harbor site near the coast off Itajaí, used as a logistical place for this industry, is a possible larvae issuer for the marine reserve. The present results corroborate this idea, since the density of larvae sparse in the nested domain is offshore, away from 1000 m isobath. Therefore, the larvae released from oil platforms situated in this area of Campos Basin during a CFE scenario are unlikely to reach the coast, Fig 13. Although, if there is a change in environmental scenarios, such as bad weather for example, the directions of currents will turn, as well as, consequently, the dispersion pattern of the larvae. The most extended trajectories are made by the ones along the shelf break, suggesting that the BC with high-speed velocities acts to increase the dispersal potential. The signal position of highly concentrated larvae in Fig 13 is similar to the one observed from the tracked eddy center, suggesting that when larvae are captured by this feature, their response is to follow the adjacent currents in which they are inserted, Fig 6.

Although the released location is on the continental shelf, the high variability in the current’s fields, typical of this region due to the meanders and eddies, acts advecting the larvae to the open ocean. The nonlinear CFE traps these organisms in a circular pathway, restricting the potential dispersal. On the other hand, considering that the longer the larvae spend in the same region, the greater the chance of colonization [81–84], this eddy increases the settlement potential in its pathway. The region where the results showed a greater larval concentration, the pre-salt zone in Santos Basin, has high density of oil platforms [85], so there are fixed structures where *Tubastraea* spp. can colonize. Thus the mesoscale features on the SE region support the absence of fluxes between the release site and the coast, but possibly collaborate to the inter-platforms genetic fluxes.

## Conclusion

The present study focused on understanding the dispersal patterns of the nonindigenous *Tubastraea* spp. species in a cyclonic eddy scenario off the Cape Frio region and in Brazil Current stable situation. The CFE was tracked using a Python algorithm that supports non-linearity analysis, translation patterns, and the identification of eddy boundaries to access which larvae are within the eddy domain. The hydrodynamic model results were consistent with the mesoscale features on the Southeastern Brazilian coast, such as the BC, the CFE, and upwelling. This study revealed that offshore oil and gas platforms in Campos Basin are not necessarily a direct source of larvae for the coastal regions of Brazil, as none of the individual larvae reached the coast, and the kernel of the highest density of these organisms stayed away of the 1000 m isobath, Figs 7–13. Then, possibly, the Brazil Current acts as a physical barrier preventing offshore planktonic organisms from reaching the coastal regions. However, the CFE possibly induces colonization between Campos and Santos basin oil platforms. The eddy captured a significant amount of larvae; therefore, these organisms stay at the mercy of the dynamics of nonlinear quasi-stationary CFE, causing the larvae inside the eddy to travel shorter distances than the ones that stayed in the axis of greater speed of the BC. In addition, the results of this research indicate that the youngest larvae may reach depths around 60 m even when emitted on the surface. Also, due to the vertical motion, there are sections in this eddy where larvae are concentrated at surface levels and others scattered in the subsurface. This possibility could be related to *in situ* observations of oil rig areas colonized by *Tubastraea* spp. in depths of about 80 m [28]. The results show that larvae that do not interact with CFE are dispersed over greater distances covering almost 600 km. On the other hand, the ones that stay longer inside the eddy do not pass 350 km from the origin site. In that regard, the cyclonic eddy off Cape Frio reduces the potential horizontal dispersion of this alien species with planktonic phase. Thus the present study brings new approaches to understanding how *Tubastraea* spp. larvae interact with the CFE and how the dispersion pattern is modified between a stability scenario, where Brazil Current is dominant, and an instability setting where mesoscale activity is pronounced. Even more, to clarify the dynamics mechanisms imposed on larvae inside a cyclonic CFE, such as downwelling. Although there was a biological approach to represent the Sun-Coral larvae, the dispersion model must be best fitted with biological aspects to better represent this invasive specie’s dispersion.
